# Regulation of Rho guanine nucleotide exchange factor 3 by phosphorylation in the PH domain

**DOI:** 10.1016/j.isci.2025.112753

**Published:** 2025-05-26

**Authors:** Jesus F. Moreno, Jae-Sung You, Carlos C. Rodriguez, Shashank Pant, Adriana Reyes-Ordoñez, Reean Abdullah, Nilmani Singh, Maxine J. van der Donk, Emad Tajkhorshid, Jie Chen

**Affiliations:** 1 Department of Cell and Developmental Biology, University of Illinois at Urbana-Champaign, Urbana, IL, USA; 2 Department of Biochemistry, University of Illinois at Urbana-Champaign, Urbana, IL, USA; 3 Theoretical and Computational Biophysics Group, NIH Resource for Macromolecular Modeling and Visualization, Beckman Institute for Advanced Science and Technology, University of Illinois at Urbana-Champaign, Urbana, IL, USA; 4 Center for Biophysics and Quantitative Biology, University of Illinois at Urbana-Champaign, Urbana, IL, USA; 5 Cancer Center at Illinois, University of Illinois at Urbana-Champaign, Urbana, IL, USA; 6 Department of Biomedical and Translational Sciences, Carle Illinois College of Medicine, University of Illinois at Urbana-Champaign, Urbana, IL, USA; 7 Department of Chemistry, Northwestern University, Evanston, IL, USA

**Keywords:** Biochemistry, In silico biology, Molecular biology

## Abstract

Although most members of the Rho guanine nucleotide exchange factor (RhoGEF) family are found to be phosphorylated, how phosphorylation regulates RhoGEF function is poorly understood. Here we report the discovery of a mechanism of RhoGEF regulation by phosphorylation. We find that ARHGEF3 is phosphorylated in the pleckstrin homology (PH) domain in a protein kinase C (PKC)-dependent manner. This phosphorylation inhibits ARHGEF3 activation of RhoA and actin stress fiber formation in the cells, and it also disrupts ARHGEF3 binding to PI(3,5)P_2_ but not to PI(4,5)P_2_. Guided by molecular dynamics simulation, a mutation in the PH domain is identified to uncouple ARHGEF3 binding to the two lipids, which is used to rule out a role of PI(3,5)P_2_ in regulating GEF activity. Results of *in vitro* GEF assays suggest that PH domain phosphorylation diminishes ARHGEF3 catalytic activity, likely through an allosteric mechanism. Our findings reveal a previously unknown type of regulatory mechanism for the family of RhoGEFs.

## Highlights

• ARHGEF3 is phosphorylated on S399 within the PH domain
• S399 phosphorylation is mediated by an nPKC
• S399 phosphorylation selectively disrupts binding to PI(3,5)P_2_ and not PI(4,5)P_2_
• S399 phosphorylation directly inhibits the GEF activity of ARHGEF3

## Graphical abstract

**Figure:**
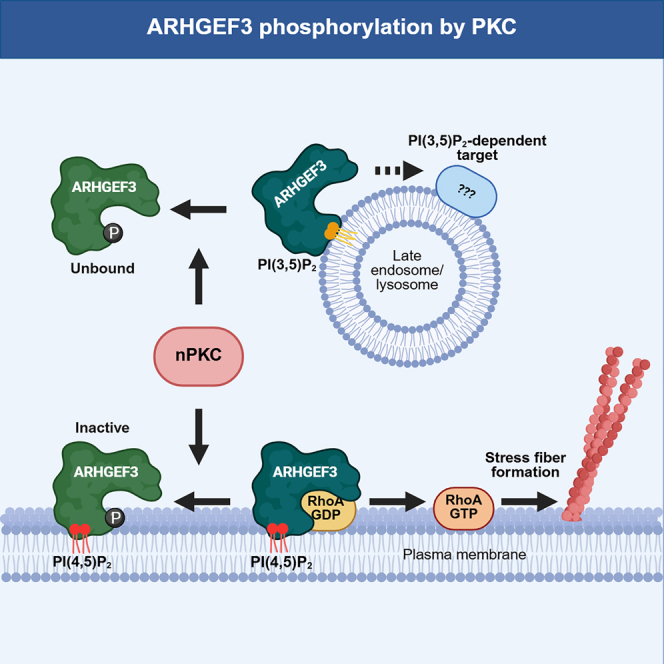

## Introduction

Rho GTPases are molecular switches that coordinate the organization of the cell’s cytoskeletal architecture. Rho proteins switch between inactive GDP-bound and active GTP-bound states, facilitated by Rho guanine nucleotide exchange factors (RhoGEFs).^1^^,^^2^^,^^3^ Members of the RhoGEF family are involved in wide-ranging biological and cellular processes, such as embryonic development, cell migration, proliferation, and survival.^4^^,^^5^ Moreover, the overexpression and mutation of RhoGEFs are linked to various disorders, including several types of cancer.^2^^,^^4^^,^^5^^,^^6^ Hence, there is a critical need to understand the mechanisms involved in the regulation of RhoGEFs.

The diffuse B-cell lymphoma (Dbl) family of RhoGEFs, containing the signature Dbl homology (DH) domain, is encoded by 71 genes in the human genome.^6^ Within this family, 67 of the 71 members have a pleckstrin homology (PH) domain immediately following the DH domain.^2^^,^^6^^,^^7^ The role of the PH domain in RhoGEFs appears to be diverse within this family of proteins, and reported mechanisms include conferring substrate selectivity, control of subcellular localization, and direct impact on catalytic activity.^1^^,^^7^^,^^8^ Although some PH domains were known to interact with phosphatidylinositol phosphates (PIPs),^9^ strong or specific interaction with PIPs was thought to be uncommon for PH domains in RhoGEFs of the Dbl family.^1^^,^^10^ However, a recent study from our laboratory revealed that a surprisingly large number of PH domain–containing proteins interact with PIPs specifically, including many RhoGEFs previously not known to bind any PIP.^11^ Therefore, specific binding to a PIP(s) may be a prevalent regulatory mechanism for RhoGEFs.

Phosphorylation is another key regulatory mechanism for RhoGEFs.^12^ For a few of these proteins (e.g., Vav1,^13^ Ephexins,^14^ and ECT2^15^), phosphorylation occurring on domains outside of DH-PH has been found to release autoinhibition and promote GEF activation. The release of autoinhibition may be further facilitated by interactions between the regulatory domains in RhoGEFs and other proteins.^14^^,^^15^^,^^16^ In the case of oncogenic P-REX1, phosphorylation on 2 non-catalytic domains has been found to modulate its binding to PIP_3_ and phosphatidic acid.^17^^,^^18^^,^^19^ Phosphorylation-mediated release of autoinhibition has also been reported for GEFs of small G proteins other than Rho, RapGEF1 (C3G) being an example.^20^ However, to date only a small number of RhoGEFs have been reported to be regulated by phosphorylation and there are even fewer examples of regulation by phosphorylation on the PH domain. Given that phosphorylation is a key mechanism of signal transduction, investigation into the regulation of any RhoGEF by phosphorylation is warranted.

ARHGEF3 is a member of the Dbl family and a GEF for RhoA and RhoB.^21^ ARHGEF3 regulates actin cytoskeleton reorganization,^21^ and it is implicated in a variety of biological processes including cell survival,^22^ mitosis,^23^ cell migration,^24^ myogenic differentiation,^22^ and autophagy in muscle regeneration and muscular dystrophy.^25^^,^^26^ The PH domain of ARHGEF3 interacts with the membrane lipids phosphatidylinositol 4,5-bisphosphate (PI(4,5)P_2_) and phosphatidylinositol 3,5-bisphosphate (PI(3,5)P_2_).^11^ PI(4,5)P_2_ binding is necessary for ARHGEF3 localization to the plasma membrane and its GEF activity,^11^ whereas the role of PI(3,5)P_2_ binding in ARHGEF3 function is not yet known. Regulation of ARHGEF3 by phosphorylation has not been reported. In the current study, we find that ARHGEF3 is phosphorylated in its PH domain, resulting in reduced GEF activity toward RhoA and diminished actin stress fiber formation in the cell. This phosphorylation also differentially affects ARHGEF3 binding to PIPs, but phosphorylation most likely exerts an allosteric effect to impact GEF activity independent of lipid binding. These findings reveal the first example of PH domain phosphorylation directly inhibiting the catalytic activity of a RhoGEF.

## Results

### PMA stimulates ARHGEF3 phosphorylation

**Figure 1:**
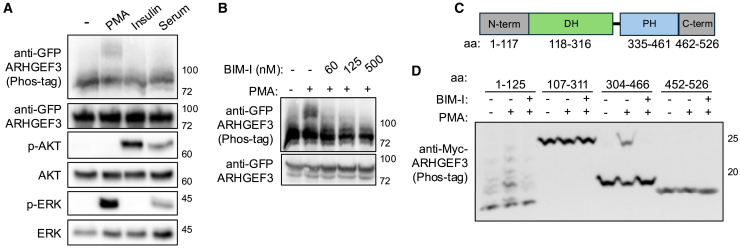
ARHGEF3 is phosphorylated on the PH domain in a PKC-dependent manner (A) HEK293 cells were transfected with GFP-ARHGEF3 for 24 h, serum-starved overnight, and then stimulated with PMA (100 nM), insulin (100 nM), or fetal bovine serum (10%) for 30 min. Cell lysates were collected and run on a Phos-tag gel (top panel) as well as regular SDS-PAGE, followed by western blotting. Recombinant ARHGEF3 proteins were detected by anti-GFP. (B) Cells were transfected and starved as in (A) and stimulated with 100 nM PMA for 30 min, followed by western analysis as in (A). BIM-1 at increasing concentrations were added to the cells 30 min before PMA addition. (C) Domain structure of ARHGEF3. The amino acid numbers at domain boundaries were predicted by AlphaFold 3. (D) Cells were transfected with Myc-tagged fragments of ARHGEF3 and subsequently treated as in (B) except that 1 μM BIM-I was used, and the lysates were analyzed on a Phos-tag gel. Molecular weight markers (kDa) are indicated on the right side of blots.

To examine whether ARHGEF3 was a phospho-protein, we performed Phos-tag gel electrophoresis with recombinant GFP-ARHGEF3 expressed in human embryonic kidney (HEK) 293 cells in response to stimulation by fetal bovine serum, insulin, and 12-*O*-tetradecanoylphorbol-13-acetate (PMA). As shown in Figure 1A, PMA stimulation caused an upshift of the protein band on the Phos-tag gel, suggesting phosphorylation of ARHGEF3. As expected, PMA stimulation led to activation of the MAP kinase ERK in these cells, whereas insulin and serum activated AKT (Figure 1A). As a structural mimetic of diacylglycerol (DAG), PMA activates protein kinase C (PKC).^27^ Indeed, bisindolylmaleimide I (BIM-I), a PKC inhibitor, blocked the PMA-stimulated ARHGEF3 band shift on Phos-tag gels in a dose-dependent manner (Figure 1B), suggesting that ARHGEF3 phosphorylation is dependent on PKC. Next, we sought to identify the PKC-dependent phosphorylation site(s) within the different domains of ARHGEF3 (Figure 1C). Four overlapping fragments of ARHGEF3 were expressed as Myc-tagged proteins and subjected to analysis on Phos-tag gels. As shown in Figure 1D, amino acid (aa) 304–466 produced a single slow-migrating band in response to PMA stimulation of the cells, which was abolished by BIM treatment. The N-terminal aa 1–125 fragment also showed multiple upshifted bands that were PMA-stimulated and BIM-I sensitive, suggesting that there may be multiple PKC-dependent phosphorylation sites in that region.

### ARHGEF3 is phosphorylated at Ser399 in an nPKC-dependent manner

**Figure 2:**
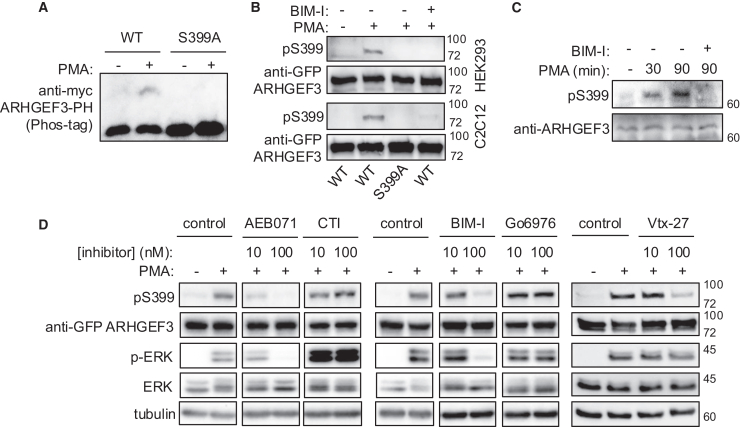
ARHGEF3 is phosphorylated in S399 by nPKCs (A) HEK293 cells were transfected with Myc-tagged ARHGEF3 PH domain (WT or S399A) for 24 h, serum-starved overnight, and then stimulated with 100 nM PMA for 30 min. Lysates were collected and run on a Phos-tag gel, followed by anti-Myc western blotting. (B) HEK293 and C2C12 cells were transfected with WT or S399A GFP-ARHGEF3 and then treated with 100 nM PMA and 1 μM BIM-I for 30 min. Lysates were analyzed by western blotting. (C) HEK293 cells were serum-starved overnight and then stimulated with 100 nM PMA for 30 or 90 min, with 1 μM BIM-I added 30 min before PMA addition. Endogenous pS399-ARHGEF3 and total ARHGEF3 were analyzed by western blotting. (D) HEK293 cells were transfected with GFP-ARHGEF3 for 24 h, pretreated with inhibitors for 10 min, and then stimulated with 100 nM PMA for 30 min, followed by cell lysis and western blotting. Inhibitor-treated bands are shown with their respective positive and negative controls from the same membrane even if they are presented separately; original images of the full blots are shown in Figure 1, Figure 2, Figure 3, Figure 4, Figure 5, Figure 6and S5. All blots shown are representative images of three or more independent experiments with similar outcomes. Molecular weight markers (kDa) are indicated on the right side of blots.

Analysis of the phosphoproteomic datasets in PhosphoSitePlus^28^ revealed that of the 67 Dbl RhoGEFs with tandem DH-PH, about two-thirds (42) were phosphorylated in the PH domain. Although most of those phosphorylation sites have not been characterized functionally, the prevalence of phosphorylation on the PH domain suggests regulatory roles. Therefore, we decided to focus on identifying the phosphorylation site(s) in the aa 304–466 fragment containing the PH domain. Within this domain, bioinformatic prediction (NetPhos-3.1: https://services.healthtech.dtu.dk/services/NetPhos-3.1/)^29^ identified PKC substrate motif surrounding Ser399. Additionally, S399 phosphorylation of endogenous ARHGEF3 also appeared in several high-throughput phosphoproteomic studies.^30^^,^^31^^,^^32^ We introduced a phospho-null mutation (S399A) within the aa 304–466 fragment, which abolished the PMA-dependent band shift (Figure 2A), suggesting that S399 was the predominant, if not the only, site phosphorylated in the PH domain.

We went on to generate a phosphopeptide-specific antibody against pS399-ARHGEF3. Transiently expressed recombinant wild-type (WT) but not S399A-ARHGEF3 was recognized by the antibody in a PMA-stimulated and BIM-sensitive manner in HEK293 cells (Figure 2B). A time course of PMA stimulation was performed, and S399 phosphorylation was detected by 5 min, peaked around 20–30 min, and persisted through 60 min (Figure S1). The phospho-S399 antibody also detected recombinant WT-ARHGEF3 in a PMA-stimulated and BIM-sensitive manner in the mouse myoblast cell line C2C12 (Figure 2B). Importantly, the endogenous ARHGEF3 protein was also detected by the anti-pS399 antibody in a PMA-stimulated and BIM-inhibited manner (Figure 2C). To establish S399 phosphorylation response beyond the DAG mimic PMA to a physiological stimulus, we treated HEK293 cells with epinephrine, known to stimulate GPCR signaling to activate PLCβ and subsequently PKC. As shown in Figure S2, epinephrine treatment consistently, albeit modestly, activated S399 phosphorylation in a rapid and transient fashion.

Because PMA also activated ERK (see Figure 1A), we tested the involvement of ERK in S399 phosphorylation using the MEK inhibitor U0126. We found that pS399 was unaffected by ERK inhibition (Figure S3). DAG (hence PMA) activates the conventional (cPKC) and novel (nPKC) subfamilies of PKCs. To identify the subfamily of PKC responsible for S399 phosphorylation, we compared the effects of five PKC inhibitors (all with IC_50_ in the nanomolar range) on PMA stimulation of pS399 in HEK293 cells. AEB071 and BIM-I inhibit both cPKC and nPKC,^33^^,^^34^ Go6976 is a selective cPKC inhibitor,^35^ and Vtx27 inhibits all nPKC isoforms.^36^ At 100 nM, which is well above the K_d_ of any of the inhibitors for their specific targets, AEB071, BIM-I, and Vtx27 all markedly inhibited S399 phosphorylation, whereas Go6976 had no effect (Figures 2D, S4 and S5). Therefore, phosphorylation of S399 in response to PMA stimulation is likely mediated by nPKC. Neither PKCδ knockdown (Figure S6) nor treatment with the PKCθ-specific inhibitor CTI (Figure 2D) affected PMA-stimulated pS399-ARHGEF3, ruling out either of these two isoforms to be solely responsible. However, it is possible that more than one nPKC may contribute to S399 phosphorylation in the cells, in which case inhibiting or knocking down any one kinase would not have an appreciable effect. We also tested those inhibitors on recombinant ARHGEF3 expressed in C2C12 cells and found that higher doses of the inhibitors were needed to inhibit pS399 in these cells compared with HEK293 (Figure S7). This difference could be due to different concentrations of endogenous PKC in these two cell lines. Under the higher concentrations of inhibitors (1 μM) needed to inhibit ARHGEF3 phosphorylation in C2C12, isoform specificity can no longer be inferred from the data.

### PKC-dependent S399 phosphorylation impairs GEF activity of ARHGEF3

**Figure 3:**
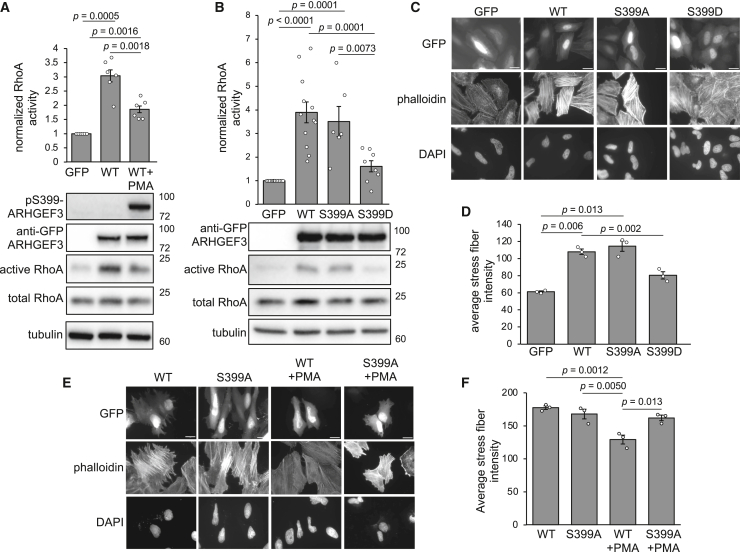
S399 phosphorylation reduces ARHGEF3 activity (A) HEK293 cells were transfected with GFP-ARHGEF3 (or GFP as control) for 24 h and then stimulated with 100 nM PMA for 1 h. Cell lysates were subjected to active RhoA pull-down by GST-Rhotekin beads and analyzed by western blotting. Western signals were quantified by densitometry, and relative RhoA activity was expressed as the ratio of active RhoA (pulldown) versus total RhoA (lysate), normalized to control as 1. Data are presented as mean ± SEM (*n* = 6). One-sample or paired t test was performed, and *p* values for significant differences (*p* < 0.05) are indicated on the graph. Representative blots are shown. pS399-GFP-ARHGEF3 western blot is also shown. Molecular weight markers (kDa) are indicated on the right side of blots. (B) Cells were transfected with WT-, S399A-, or S399D-GFP-ARHGEF3 (or GFP as control), followed by active RhoA pulldown and quantification as in (A) (*n* ≥ 5). Linear mixed-model analysis followed by a Tukey test was performed for pairwise comparison, and *p* values for significant differences (*p* < 0.05) are indicated on the graph. Representative blots are shown. (C) HeLa cells were transfected with WT-, S399A-, or S399D-GFP-ARHGEF3 (or GFP as control) followed by fixation and phalloidin/DAPI staining. Representative images of three independent experiments are shown (scale bar, 2 μm). (D) Fluorescence intensities of stress fibers were quantified for the experiments in (C). Data are shown as mean ± SEM (*n* = 3 experiments, >20 cells per experiment). Paired t test was performed, and *p* values for significant differences (*p* < 0.05) are indicated on the graph. (E) HeLa cells were transfected with WT- or S399A-GFP-ARHGEF3 for 24 h and then stimulated with 100 nM PMA for 1 h, followed by fixation and phalloidin/DAPI staining. Representative images of three independent experiments are shown (scale bar, 2 μm). (F) Fluorescence intensities of stress fibers were quantified for the experiments in (E). Data are shown as mean ± SEM (n = 3 experiments, >25 cells per experiment). Two-way ANOVA was performed followed by Tukey test. Significant effect was found with PMA treatment but not with mutation, and *p* values for significant differences (*p* < 0.05) are indicated on the graph.

To probe the functional relevance of S399 phosphorylation, we examined the GEF activity of ARHGEF3 under PKC activation in cells. A RhoA pull-down assay was performed to assess cellular RhoA activity. The expression of WT ARHGEF3 markedly elevated the level of active RhoA, and this activity was significantly reduced by PMA stimulation with concomitant phosphorylation on S399 (Figure 3A). Since PMA stimulation also promoted phosphorylation of the N terminus of ARHGEF3, we assessed the specific contribution of pS399 to GEF activity using the phospho-mimetic (S399D) and phospho-null (S399A) mutants of ARHGEF3. As shown in Figure 3B, S399D-ARHGEF3 displayed significantly reduced GEF activity compared with WT, whereas the S399A mutant had no effect.

Next, we examined actin stress fiber formation as a biological consequence of ARHGEF3 activation of RhoA. As shown in Figure 3C, overexpression of ARHGEF3 induced actin stress fiber formation in HeLa cells as expected, and S399A-ARHGEF3 had a similar effect. On the other hand, S399D-ARHGEF3 exhibited a reduced ability to induce stress fibers. Quantification of the intensity of F-actin is shown in Figure 3D. Consistent with PKC phosphorylation of S399, PMA stimulation decreased the formation of stress fibers in WT-ARHGEF3- but not S399A-ARHGEF3-expressing cells (Figures 3E and 3F). Taken together, our results strongly suggest that PKC-dependent S399 phosphorylation negatively regulates ARHGEF3 GEF activity toward RhoA. Notably, GFP-ARHGEF3 was found in both the cytoplasm and the nucleus, consistent with a previous report of ARHGEF3 subcellular localization.^23^ However, a nuclear function of ARHGEF3 is not known, and we previously reported that the GEF activity of ARHGEF3 requires its interaction with PI(4,5)P_2_ at the plasma membrane.^11^

### S399 phosphorylation impairs ARHGEF3 binding to PI(3,5)P_2_

**Figure 4:**
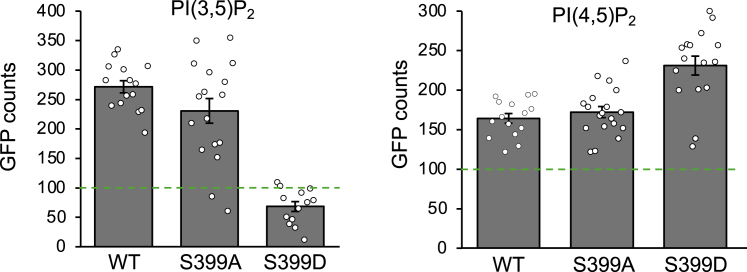
S399 phosphorylation impairs ARHGEF3 binding to PI(3,5)P_2_ HEK293 cells were transfected with WT-, S399A-, or S399D-GFP-ARHGEF3 for 24 h, and cell lysates were subjected to lipid SiMPull assays using small unilamellar vesicles (SUVs) containing 5% PI(3,5)P_2_ or PI(4,5)P_2_. Data shown are mean ± SEM for one experiment (*n* ≥ 10 images). Three independent experiments were performed with similar outcomes. The threshold of 100 GFP counts (pulled down by SUVs), previously determined as a detectable interaction,^11^ is indicated by dotted lines.

The location of S399 in the PH domain prompted us to investigate whether this phosphorylation influences ARHGEF3 binding to PI(4,5)P_2_ and PI(3,5)P_2_. We performed lipid single-molecule pull-down (lipid-SiMPull) assay^11^^,^^37^ to examine ARHGEF3 interaction with PIPs. WT, S399A, and S399D GFP-ARHGEF3 were transiently expressed in HEK293 cells, and whole-cell lysates were subjected to SiMPull assays using small unilamellar vesicles containing either PI(4,5)P_2_ or PI(3,5)P_2_. As shown in Figure 4, the phospho-null (S399A) mutant of ARHGEF3 behaved similarly to the WT protein in binding to both PIPs. On the other hand, the phospho-mimetic mutant (S399D) bound only PI(4,5)P_2_ and not PI(3,5)P_2_. This observation suggests that S399 phosphorylation of ARHGEF3 selectively blocks PI(3,5)P_2_ binding.

### PI(3,5)P_2_ is dispensable for GEF activity of ARHGEF3

Since S399 phosphorylation impaired both GEF activity and PI(3,5)P_2_ binding of ARHGEF3 as suggested by our data with the mutants, it was formally possible that S399 phosphorylation inhibited GEF activity by disrupting PI(3,5)P_2_ binding. To test this hypothesis, we began by examining whether PI(3,5)P_2_ binding was necessary for ARHGEF3 GEF activity. To do so, we needed to uncouple ARHGEF3 binding to PI(3,5)P_2_ and PI(4,5)P_2_ because the latter has already been shown to be necessary for ARHGEF3 GEF activity.^11^ A mutant that selectively disrupts PI(3,5)P_2_ binding while preserving PI(4,5)P_2_ binding of ARHGEF3 would serve that purpose.

**Figure 5:**
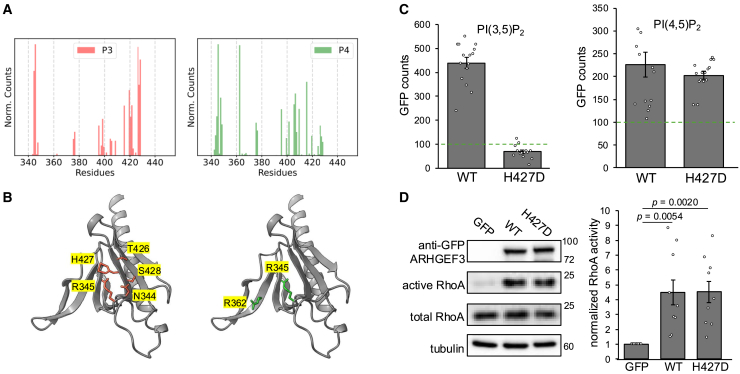
H427 distinguishes PH domain interaction with PI(3,5)P_2_ and PI(4,5)P_2_ (A) MD simulations were performed with the ARHGEF3 PH domain bound to membrane bilayers containing PI(4,5)P_2_ or PI(3,5)P_2_. The histograms show normalized ensemble-averaged number of contacts formed between residues in the PH domain and the 3- and 4-phosphate groups (3P and 4P) of PI(3,5)P_2_ and PI(4,5)P_2_, respectively. Data were averaged over all simulation replicas for each lipid composition. (B) ARHGEF3 PH domain, and highlighted on the ribbon structure are the residues with the highest frequency of interaction with 3P or 4P. (C) HEK293 cells were transfected with WT- or H427D-GFP-ARHGEF3 for 24 h, and cell lysates were subjected to lipid SiMPull assays as in Figure 4. Data shown are mean ± SEM for one experiment (*n* ≥ 10 images). Three independent experiments were performed with similar outcomes. Previously determined threshold for binding (100 GFP counts) is indicated by dotted lines. (D) HEK293 cells were transfected with WT or H427D-GFP-ARHGEF3 (or GFP as control) for 24 h. Cell lysates were subjected to active RhoA pulldown by GST-Rhotekin beads and analyzed by western blotting. Western signals were quantified by densitometry, and relative RhoA activity was expressed as the ratio of active RhoA (pulldown) versus total RhoA (lysate), normalized to control as 1. Data are presented as mean ± SEM (*n* = 9). One-sample or paired t test was performed, and *p* values for significant differences (*p* < 0.05) are indicated on the graph. Representative blots are shown. Molecular weight markers (kDa) are indicated on the right side of blots.

Previously, we used all-atom molecular dynamics (MD) simulation to gain mechanistic insights into PH domain interactions with PIPs.^38^^,^^39^ Here, we set out to identify specific amino acids in the ARHGEF3 PH domain that may selectively interact with PIPs taking advantage of the predictive power of MD simulation. MD simulations using a highly mobile membrane mimetic model^40^^,^^41^^,^^42^ were performed for the PH domain of ARHGEF3 with membranes composed of phosphatidylcholine (60%), cholesterol (20%), phosphatidylserine (15%), and either PI(4,5)P_2_ or PI(3,5)P_2_ (5%), spanning a total of 20 replicas for each membrane model. Contact probability analysis of the 3- and 4-phosphate groups shows that the PH domain might utilize different amino acids for interaction with PI(3,5)P_2_ and PI(4,5)P_2_ (Figure 5A). Two groups of residues with the highest probability of contacting the 3-phosphate group of PI(3,5)P_2_ were aa 344–345 and aa 426–428 (Figure 5B). Within these groups, R345 and H427 were more likely to have strong interactions with the phosphate group due to their positive charges. R345, but not H427, was also found to have a high probability of interacting with the P4 of PI(4,5)P_2_ (Figure 5A). Interestingly, we had previously found that the R345D/H427D double mutations eliminated both PI(3,5)P_2_ and PI(4,5)P_2_ binding to ARHGEF3.^11^ Therefore, we reasoned that H427D alone might have a selective effect on the binding of the two PIPs. We introduced the H427D mutation in full-length ARHGEF3 and performed lipid-SiMPull assays. Indeed, we observed that H427D abolished PI(3,5)P_2_ binding without affecting ARHGEF3 binding to PI(4,5)P_2_ (Figure 5C).

With the PI(4,5)P_2_ and PI(3,5)P_2_ binding activities of ARHGEF3 uncoupled by H427D, we then examined the effect of this mutation on GEF activity. As shown in Figure 5D, H427D-ARHGEF3 activated RhoA in the cell to the same extent as the WT protein, suggesting that PI(3,5)P_2_ binding is dispensable for ARHGEF3 GEF activity. These results rule out the possibility that the effect of S399 phosphorylation on ARHGEF3 GEF activity is through the loss of PI(3,5)P_2_ binding.

### S399 phosphorylation directly inhibits the catalytic activity of ARHGEF3

**Figure 6:**
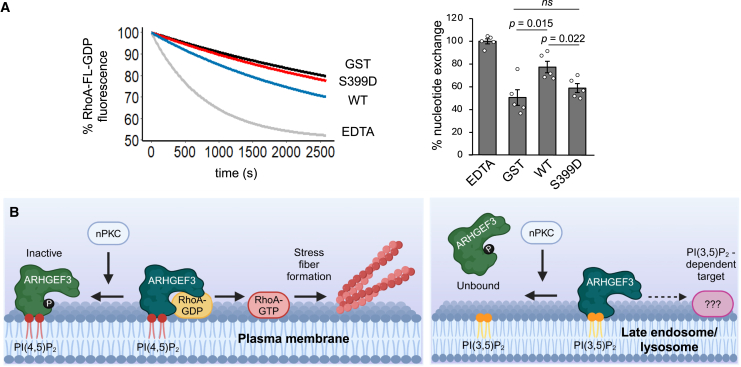
S399D reduces the catalytic activity of ARHGEF3 (A) Purified WT- and S399D-GST-ARHGEF3 were subjected to *in vitro* RhoA guanine nucleotide exchange assays. EDTA: positive control for complete nucleotide dissociation. Data from five independent experiments with similar outcomes were fit as a single exponential decay curve (left panel). RhoA nucleotide exchange activity at t = 45 min is shown on the bar graph (right panel), normalized to EDTA as 100%. Data are shown as mean ± SEM (*n* = 5). Student’s t test was performed. (B) A model of ARHGEF3 regulation. Left panel: ARHGEF3 activates RhoA at the plasma membrane, requiring binding to PI(4,5)P_2_.^11^ PKC-dependent phosphorylation in the PH domain allosterically inhibits the GEF activity of ARHGEF3, dampening RhoA activation and actin stress fiber formation. Right panel: the same phosphorylation also disrupts ARHGEF3 binding to PI(3,5)P_2_ (presumably in the late endosome), the role of which is yet to be determined. The graphics were created using BioRender.

Finally, we considered the possibility that S399 phosphorylation may directly inhibit the catalytic activity of ARHGEF3. *In vitro* GEF assays were performed with RhoA preloaded with boron-dipyrromethene (BODIPY)-GDP, and excess GTP was used for the exchange. WT and S399D ARHGEF3 proteins were expressed as GST fusion proteins and purified from bacteria. As a positive control, the chelator EDTA was added to the reaction to fully dissociate GDP. WT-ARHGEF3 displayed activity toward RhoA as expected, whereas the S399D mutant had significantly reduced nucleotide exchange activity compared with WT (Figure 6A). These results suggest that PKC-dependent phosphorylation of the PH domain allosterically inhibits the GEF activity of ARHGEF3 (Figure 6B).

## Discussion

In this study, we have identified nPKC-dependent phosphorylation in the PH domain of ARHGEF3 and a direct effect of S399 phosphorylation on inhibiting the GEF activity of ARHGEF3. Although PH domain phosphorylation occurs in the majority of RhoGEFs in the publicly available phosphoproteomic databases, very few of them have been characterized for the functional relevance or biochemical mechanism of the phosphorylation. P-REX1 is phosphorylated by PKC in its PH domain leading to decreased Rac1 activation in cells,^43^ but whether this phosphorylation directly impacts the catalytic activity of P-REX1 has not been reported. ARHGEF1 is the only RhoGEF reported to be directly *activated* by phosphorylation in the PH domain.^44^ Our current findings represent the first example of PH domain phosphorylation *inhibiting* intrinsic GEF activity of a RhoGEF, possibly through an allosteric mechanism. Aside from ARHGEF1 being phosphorylated on a tyrosine as opposed to a serine on ARHGEF3, it is also noteworthy that the steady-state activity of ARHGEF3 is higher than that of ARHGEF1 as reported by Müller et al.^23^ Hence, ARHGEF1 is most likely autoinhibited like many other RhoGEFs^23^ and the phosphorylation may release the autoinhibition. On the other hand, ARHGEF3 is one of the few RhoGEFs with a high basal activity, and its inhibition by upstream signals may serve to maintain homeostasis of RhoA activation. Future structural studies complemented by the predictive power of MD simulation and the readily available phosphoproteomic information should accelerate our understanding of RhoGEF phosphorylation and reveal new paradigms of regulation for this large family of signaling proteins.

Regulation of RhoGEFs is a key step in the timely and effective activation of RhoGTPases to control actin cytoskeleton reorganization. The PKC family of kinases are known regulators of the actin cytoskeleton and cell migration,^45^ but how PKC regulates RhoGEF activity is poorly understood. Here we show that nPKC-dependent phosphorylation of ARHGEF3 dampens actin stress fiber formation (Figure 3C). Since ARHGEF3 regulates cell migration,^24^ our findings suggest that PKC modulation of ARHGEF3 activity could be involved in coordinating RhoA-dependent actin cytoskeletal reorganization during cell migration. This possibility should be probed in future studies. The exact isoform of nPKC responsible for this regulation is most likely cell type dependent.

Phosphorylation of S399 selectively disrupts ARHGEF3 interaction with PI(3,5)P_2_, presumably in the late endosomal membrane where this lipid is found, without affecting PI(4,5)P_2_ binding (Figure 6B). Although a functional consequence of ARHGEF3-PI(3,5)P_2_ interaction remains unclear, our study is the first to identify phosphorylation in the PH domain of a RhoGEF that differentially affects interaction with two PIPs, with both structural and regulatory implications. The loops connecting the β-sheets of the PH domain are thought to be involved in the interaction with lipids.^11^^,^^46^ S399 resides within the 23-aa β5-β6 loop, flanked by two arginines (R395 and R401) as a part of PKC substrate motif,^47^ which could be involved in interacting with PIPs. The overlap between the PKC substrate motif and the PIP-binding site raises the compelling possibility that PKC-mediated phosphorylation might regulate PIP binding in other PH domain-containing proteins beyond the RhoGEF family. We can also speculate on a widespread involvement of basic amino acids-directed protein kinases (such as the AGC family to which PKC belongs) in the regulation of PH domain binding to PIPs, which can be explored through motif analysis and accurate PH domain loop predictions now available by AlphaFold.^48^

Interestingly, we also found the N terminus of ARHGEF3 to be phosphorylated on multiple sites in a PKC-dependent manner. Yet, S399 phosphorylation alone appears to be responsible for the regulation of GEF activity and stress fiber formation in response to PMA stimulation. This aligns with our previous report that ARHGEF3 functions non-canonically as an endogenous inhibitor of mammalian target of rapamycin complex 2 (mTORC2) and that the N terminus is responsible for this function, whereas the GEF activity is dispensable.^22^ A potential role of N-terminal phosphorylation on mTORC2 regulation warrants future exploration. Of note, mTORC2 regulates the stability and/or activity of cPKCs and atypical PKCs as well as at least one nPKC (PKCε).^49^^,^^50^^,^^51^ PKC phosphorylation of ARHGEF3 in the N terminus raises the intriguing possibility of a feedback regulation between mTORC2 and PKC that will also be worthy of future investigation.

### Limitations of the study

Most of our experiments to promote ARHGEF3 phosphorylation at S399 relied on robust stimulation by the synthetic DAG mimic PMA. Although we showed that a physiological agonist of GPCR signaling, epinephrine, modestly stimulated pS399 in HEK293 cells, these cells are not typically considered biological targets of epinephrine. Future studies are warranted in cellular or organismal contexts where biological stimulation and functional consequence of PKC phosphorylation of S399-ARHGEF3 can be examined.

## Resource availability

### Lead contact

Further information and requests for resources and reagents should be directed to and will be fulfilled by the lead contact, J.C. (jiechen@illinois.edu)

### Materials availability

Reagents generated by this study are available from the lead contact with a completed Materials Transfer Agreement.

### Data and code availability

• Original data are available from the lead contact upon request.
• Codes used in this study are deposited on GitHub or Zenodo. smFRET package used for SiMPull image acquisition: https://github.com/Ha-SingleMolecule Lab. IDL scripts used to process raw image files: https://doi.org/10.5281/zenodo.4925617. An example script used for MD simulation analysis: https://github.com/jesusfm2/NAMD_HMMM_code_ARHGEF3.
• Any additional information required to reanalyze the data reported in this paper is available from the lead contact upon request.

## Acknowledgments

This work was supported by grants from the National Institutes of Health: R01 GM089771 to J.C. and P41-GM104601 and R24-GM145965 to E.T. for the simulation experiments. Simulations were performed using allocations on National Science Foundation (NSF) Supercomputing Centers (ACCESS grant number MCA06N060) and Delta advanced computing and data resource, which is supported by the NSF (award OAC 2005572) and the State of Illinois.

## Author contributions

Conceptualization, J.F.M., J.-S.Y., E.T., and J.C.; investigation, J.F.M., J.-S.Y., C.C.R., S.P., A.R.-O., R.A., N.S., and M.J.v.d.D.; formal analysis, J.F.M., J.-S.Y., C.C.R., and A.R.-O.; writing – original draft, J.F.M. and J.C.; writing – review and editing, J.F.M., J.-S.Y., C.C.R., S.P., A.R.-O., R.A., E.T., and J.C.; funding acquisition, E.T. and J.C.

## Declaration of interests

The authors declare that the research was conducted in the absence of any commercial or financial relationships that could be construed as a potential conflict of interest. S.P. is currently an employee of Eli Lilly and is a shareholder of stock in Eli Lilly and Co.

## STAR★Methods

### Key resources table

**Table:** 

REAGENT or RESOURCE	SOURCE	IDENTIFIER
**Antibodies**		

GFP	Proteintech	66002-1-Ig
α-tubulin	Proteintech	66031-1-Ig; RRID: AB_11042766
AKT	Cell Signaling Technology	9272S; RRID: AB_329827
pAKT (S473)	Cell Signaling Technology	9271S; RRID: AB_329825
PKCδ	Cell Signaling Technology	2058S; RRID: AB_10694655
ERK	Cell Signaling Technology	4696S; RRID: 390780
pERK1/2 (T202/Y204)	Cell Signaling Technology	4370S; RRID: 2315112
Myc clone 9E10	Covance	Cat# MMS-150P-200; RRID: AB_291323
RhoA	Cell Signaling Technology	2117S; RRID: AB_10693922
pS399-ARHGEF3	Fabgennix International Inc	Custom-made (this paper)
ARHGEF3	Proteintech	Custom-made^22^

**Bacterial and virus strains**		

BL21	New England Biolabs	C2527

**Chemicals, peptides, and recombinant proteins**		

PMA	EMD Millipore	Cat# 524400
BIM-I	Sigma-Aldrich	Cat# 203290
AEB071	Selleck Chemicals	Cat# S2791
Go6976	Sigma-Aldrich	Cat# 365250
Vtx-27	Cayman Chemicals	Item No. 36938
CTI	Selleck Chemicals	Cat# S6577
U0126	EMD Millipore	662005
GST-Rhotekin beads	Cytoskeleton Inc	Cat# RT02
DAPI	Thermo Fisher Scientific	Cat# 62247
Rhodamine-Phalloidin	Cytoskeleton Inc	Cat# PHDR1
PEI	Sigma-Aldrich	Cat# 408727
Lipofectamine 3000	Thermo Fisher Scientific	Cat# L3000001
Phosphatidylcholine	Avanti Research	CAS 850457C
Phosphatidylserine	Avanti Research	CAS 840034P
Cholesterol	Avanti Research	CAS 700100P
PI(4,5)P_2_	Avanti Research	CAS 840046X
PI(3,5)P_2_	Avanti Research	CAS 850154P
mPEG	Laysan Bio Inc	MPEG-SVA-5000
Biotin-PEG	Laysan Bio Inc	Biotin-PEG-SVA-5000
Biotin-PE	Avanti Research	CAS 384835-52-3
Neutravidin	Thermo Fisher Scientific	PI31000
DiD	Thermo Fisher Scientific	D307
Recombinant RhoA	Cytoskeleton Inc	Cat# RH01
BODIPY-GDP	Thermo Fisher Scientific	Cat# G22360

**Experimental models: Cell lines**		

HEK293	ATCC	CRL-1573
HeLa	ATCC	CCL-2
C2C12	ATCC	CRL-1772
HEK293T	ATCC	CRL-3216

**Recombinant DNA**		

Plasmid: myc-ARHGEF3 1-125	Khanna et al.^22^	NA
Plasmid: myc-ARHGEF3 107-311	Khanna et al.^22^	NA
Plasmid: myc-ARHGEF3 304-466	Khanna et al.^22^	NA
Plasmid: myc-ARHGEF3 452-526	Khanna et al.^22^	NA
Plasmid: myc-ARHGEF3 PH	Khanna et al.^22^	NA
Plasmid: GFP-ARHGEF3	Singh et al.^11^	NA
Plasmid: GFP-S399A	This paper	NA
Plasmid: GFP-S399D	This paper	NA
Plasmid: GFP-H427D	This paper	NA
Plasmid: GST-ARHGEF3	Khanna et al.^22^	NA
Plasmid: GST-S399D	This paper	NA
Plasmid: pGEX-2T GST	Khanna et al.^22^	NA
Plasmid: psPAX2	Addgene	Plasmid #12260
Plasmid: pMD2.G	Addgene	Plasmid #12259
Plasmid: shScramble	Addgene	Plasmid # 1864
Plasmid: shPKCδ	Sigma-Aldrich	TRCN0000010193

**Software and algorithms**		

ImageJ	National Institute of Health	https://imagej.nih.gov/ij
R studio	Rstudio	http://www.rstudio.com/
Python	Python Software foundation	https://www.python.org9
NAMD	UIUC	https://www.ks.uiuc.edu/Research/namd/
SHAKE	Ryckaert et al.^52^	NA
SETTLE	Miyamoto et al.^53^	NA
SiMPull image acquisition		https://github.com/Ha-SingleMoleculeLab
LDL script		https://doi.org/10.5281/zenodo.4925617
MD simulation		https://github.com/jesusfm2/NAMD_HMMM_code_ARHGEF3.

### Experimental model and study participant details

#### Cell culture

The cells lines HEK293, HEK293T, HeLa and C2C12 were purchased from the American Type Culture Collection (ATCC, USA). The human cell lines were cultured using high-glucose (4.5g/L) DMEM with 10% fetal bovine serum (10% inactivated fetal bovine serum for HEK293T cells), 2 mM L-glutamine, and penicillin/streptomycin at 37 C in 5% CO_2_. For C2C12 cells were grown in the same medium as above but supplemented with 4 mM L-glutamine in 7.5% CO_2_.

### Method details

#### Cell transfection, stimulation and inhibitor treatment

For experiments in HEK293, cells were transfected for 16–24 h in 12- or 6-well plates using Polyethylenimine (PEI; 3 μg/μg of DNA). The DNA:PEI complex was incubated at room temperature for 15 min before adding to the cells. For experiments in C2C12 and HeLa, cells were transfected for either 24 or 48 hr with Lipofectamine™ 3000 following manufacturer’s recommendations. PMA stimulation was performed at 100 nM under different time points specified in figure legends. Epinephrine stimulation was performed on serum-starved cells at 10 μM for 2.5, 5, 10, and 20 min. For inhibitor experiments, cells were treated with the MEK inhibitor (U0126) at 5 μM or a PKC inhibitor (AEB071, BIM-I, Go6976, Vtx-27 or CTI) at 10, 100, or 1000 nM for 10 min prior to PMA stimulation.

#### Lentiviral packaging and viral transduction

HEK293T cells were transfected for 24 h with shScramble or shPKCδ DNA, psPAX2 and pMD2.G at a 10:1:9 ratio. Cell medium containing the lentivirus were collected 2 and 3 days after transfection, combined, and stored as small aliquots at -80^o^C. Cells supplemented with polybrene (8 μg/mL) were transduced with virus for 24 h and selected for 4 days in puromycin (1.5 μg/mL).

#### Western blotting and Phostag gel analysis

Cells were lysed in SDS sample buffer containing 5% β-mercaptoethanol and heated for 5 min at 95^o^C. Proteins were resolved by SDS-PAGE and transferred onto polyvinylidene fluoride membrane (EMD Millipore, Darmstadt, Germany), followed by incubation with various antibodies in accordance with manufacturers’ recommendations. Detection of horseradish peroxidase-conjugated secondary antibodies was performed with SuperSignal West Pico PLUS Chemiluminescent Substrate (Thermo Fisher Scientific, Waltham, MA, USA), and images were captured on an iBright CL1000 imaging system (Thermo Fisher Scientific, Waltham, MA, USA). Phos-tag gels of 7.5 and 12 % polyacrylamide were composed of 25 μM Phos-tag and 100 μM MnCl_2_, and protein samples were run with a constant current of 30 mA for 4.5 hr. After electrophoresis, the gels were washed in transfer buffer containing 1 mM EDTA for 15 min, followed by washing with plain transfer buffer for 15 minutes to remove EDTA. Transfer of proteins to PVDF membranes was performed with constant current of 250 mA for 100 min followed by immunoblotting. Quantification of western blots was performed by densitometry using ImageJ.

#### Active RhoA pulldown assay

To measure cellular RhoA activity, HEK293 cells were seeded at 60-80% confluency in 6-well plates, transfected for 24 hr, and then lysed with 50 mM Tris (pH 7.4), 10 mM MgCl_2_, 500 mM NaCl, 1% NP-40 and 1x Protease inhibitor cocktail. To assess active RhoA, cell lysates were incubated for 1 hr at 4^o^C with agarose beads containing 30 μg of GST-rhotekin (Cytoskeleton Inc., Denver, CO) followed by a 500 μL wash with 50 mM Tris (pH 7.4), 10 mM MgCl_2_, 150 mM NaCl, 1% NP-40 and 1x Protease inhibitor cocktail. The beads were resuspended in SDS sample buffer and heated at 95^o^C for 5 min, followed by analysis by western blotting of RhoA. Western signals were quantified by densitometry, and RhoA activation was represented by the ratio of active versus total RhoA.

#### Fluorescence imaging

HeLa cells seeded on gelatin (0.2%)-coated glass coverslips were fixed in 2% paraformaldehyde at room temperature for 15 min, permeabilized with 0.5% Triton X-100 for 5 min, and incubated with 600 nM rhodamine-phalloidin and 3% bovine serum albumin in PBS at room temperature for 30 min, followed by incubation with DAPI (1 μg/μL) for 10 min. Imaging was performed with a Leica DMI 4000B fluorescence microscope (Leica, Wetzlar, Germany) using a 63x/1.40 oil lens (HCX PL APO, Leica, Germany). Images were captured with a RETIGA EXi camera (QImaging, Surry, BC, Canada) and processed with Image Pro Express software (Media Cybernetics, Rockville, MD, USA). The average intensity of actin stress fibers was quantified using Fiji in ImageJ (NIH). For dense fiber bundles, a single line was quantified for each bundle within a cell.

#### Lipid vesicle preparation

Small unilamellar vesicles (SUVs) were generated using bath sonication as previously described.^11^ Briefly, a total of 0.166 μmol lipids were mixed in chloroform, dried under nitrogen flow, and resuspended in 100 μL of vesicle buffer (10 mM Tris-HCl, pH 8.0, 150 mM NaCl) to a final concentration of 1.66 mM. The lipid composition of the SUVs was 60 mol%. Phosphatidylcholine, 15 mol% phosphatidylserine, 20 mol% cholesterol, and 5 mol% PI(4,5)P_2_ or PI(3,5)P_2_. Following bath sonication (Laboratory Supplies, Hickscile, NY, model G112SPIT, 600 v, 80 kc, and 0.5 A), the SUVs were collected as supernatant after ultracentrifugation at 194,398xg in a TLA100.3 rotor for 1 hour at 25^o^C.

#### Cell lysis for lipid-SiMPull assay

HEK293 cells were seeded in 6-well plates and transfected at a 60 -70% confluence using 2 μg plasmid DNA and PEI as described above. After 16-24 hr, cells were washed once using ice-cold PBS and each well was re-suspended in 250 μL of detergent-free lysis buffer (40 mM HEPES pH 8.0, 150 mM NaCl, 10 mM ß-glycerophosphate, 10 mM sodium pyrophostate, 2 mM EDTA, 1x Sigma protease inhibitor cocktail). Cells were lysed by probe-sonication for 3 seconds on ice followed by ultracentrifugation at 90,000xg for 30 minutes at 4°C. EGFP concentration in lysates was measured in a plate reader (Agilent, BioTek Synergy LX) at excitation 488/emission 520, and calculated using a standard curve of pure recombinant EGFP (ProSpecbio #PRO-1606). Each cell lysate was diluted in lysis buffer to 5 nM EGFP for the assays.

#### Lipid-SiMPull assay

Quartz slides were prepared as previously described.^11^^,^^37^ Briefly, slides were cleaned and passivated with PEG doped with 0.10.2% biotin-PEG. Just prior to use in the assay, the slides were divided into chambers, incubated with NeutrAvidin (200 μg/mL) for 10 min, followed by a 10 min incubation with biotinylated SUVs. Untethered SUVs were washed out of the slide chamber using vesicle buffer (10 mM Tris·HCl, pH 8.0, 150 mM NaCl). Freshly prepared whole-cell lysates (80 μL) were flowed into slide chambers and incubated for 15 min at room temperature. An inverted total internal reflection fluorescence (TIRF) microscope with Olympus 100x, NA 1.4 lens, and EMCCD camera (Andor iXon Ultra 897) was used to acquire single-molecule data at 10 frames/second. TIRF images were acquired, processed, and quantified as previously described.^11^ For each image the background number of GFP spots was measured prior to lysate incubation and subtracted from GFP spots after lysate addition. At least 10 SiMPull images (1600 μm^2^ each) were analyzed to generate the average number of GFP spots per imaging area for each assay. The threshold for specific interaction had been previously determined to be 100 GFP spots based on mean of known negative controls plus 2x standard deviation.^11^

#### Molecular dynamics simulations

The HMMM model accelerates membrane rearrangements and sampling of lipid-protein interactions by employing an organic solvent region, 1,1-dichloroethane (DCLE), that mimics the membrane’s hydrophobic core, thereby facilitating more thorough exploration of protein-lipid contacts.^40^^,^^41^^,^^42^ Two sets of membrane compositions were considered: (1) 60% POPC, 20% cholesterol, 15% POPS, and 5% PI(3,5)P_2_; and (2) 60% POPC, 20% cholesterol, 15% POPS, and 5% PI(4,5)P_2_. These lipid compositions were chosen to reflect physiologically relevant conditions while allowing direct comparison of ARHGEF3 PH’s interaction with distinct phosphatidylinositol bisphosphates. The ARHGEF3 PH domain was initially placed approximately 25 Å above the membrane surface, ensuring no bias from direct membrane contact at the start. For each membrane composition, 20 independent replicas were generated, each with a unique initial protein orientation to enhance conformational sampling and reduce orientation-specific artifacts. Each replica was simulated for 100 ns. Coordinates and topologies were generated and assembled using standard molecular modeling tools, and initial energy minimization was performed to relieve unfavorable contacts. All simulations were conducted using NAMD^54^^,^^55^ with the CHARMM36m force field for proteins and the CHARMM36 force field for lipids. The systems were solvated using the TIP3P water model,^56^^,^^57^ and appropriate ions were added to achieve electroneutrality and an ionic concentration of ∼150 mM NaCl. Nonbonded interactions were computed using a cutoff of 12 Å with a switching function applied at 10 Å to smoothly truncate the van der Waals interactions. Long-range electrostatics were calculated with the particle mesh Ewald (PME) method,^58^ providing accurate treatment of Coulombic interactions. The temperature was maintained at 310 K using a Langevin thermostat with a damping coefficient of 1.0 ps^−1^, and the pressure was held at 1 bar using the Nosé–Hoover Langevin piston method.^59^ The simulation cell dimensions were allowed to fluctuate independently in three dimensions, enabling the system to achieve its natural equilibrium density, while the x/y ratio of the membrane plane was kept constant to maintain membrane integrity.

Covalent bonds to hydrogen atoms were constrained using the SHAKE^52^ and SETTLE^53^ algorithms, allowing a 2 fs integration timestep. This setup enabled stable long-timescale simulations with efficient sampling of protein and lipid dynamics. Following initial equilibration protocols, each production simulation was continued for 100 ns, providing sufficient sampling to identify residue-level interactions and differences in lipid engagement between PI(3,5)P_2_ and PI(4,5) P2-containing membranes.

Trajectory frames were analyzed using the MD Analysis Python library^60^^,^^61^ in combination with custom Python scripts. To focus on relevant interaction events, we first identified time segments within the replicate simulations where stable binding of the ARHGEF3 PH domain to the membrane was observed. Stable binding was defined as continuous protein-lipid contact maintained over multiple consecutive frames. Within these stable binding intervals, residue-level contacts were quantified by measuring the distance between heavy atoms in candidate side chains of the ARHGEF3 PH domain and the phosphate group characteristic of each of the PIPs assessed, namely, the 3-phosphate of PI(3,5)P_2_ and the 4-phosphate of PI(4,5)P_2_. A 3.5 Å distance cutoff was used to define a contact for performed residue-PIPs contact analysis.^38^^,^^39^

#### Protein expression and purification in *E. coli*

The BL21 *E. coli* strain was used for protein expression. GST-ARHGEF3 and GST-ARHGEF3-S399D in pGEX-4T1 were expressed with 0.3 mM IPTG induction at 18°C overnight. After induction, cells from 1 L culture were resuspended in 20 mL PBS with 1 mg/mL lysozyme and frozen at -20°C. Bacteria were spontaneously lysed upon thawing, and the lysates were cleared through sonication and centrifugation at 4°C. Lysates were incubated with 300 μL of glutathione Sepharose beads (GE Healthcare) for 1 hr at 4°C followed by packing in a column and washed with 3 mL of PBS. Proteins were eluted with 100 mM Tris-HCl, pH 8.0, 10 mM glutathione. The elutions were mixed with freezing buffer (25 mM Tris-HCl, pH 7.4, 50% glycerol) at 1:1, followed by concentration using centrifugal filters (Amicon® Ultra, Ultracel-50K). Aliquots were stored at -80°C. Concentration of the protein was determined through densitometry after Coomassie blue staining using a standard curve of BSA.

#### *In vitro* RhoA guanine nucleotide exchange assay

The nucleotide exchange activity of RhoA was measured *in vitro* as the dissociation of BODIPY-GDP from RhoA in the presence of excess GTP. Recombinant RhoA was preloaded with BODIPY-GDP (Thermo Fisher Scientific, Waltham, MA) at a 1:1 ratio in nucleotide loading buffer (20 mM Tris-HCl pH 7.5, 100 NaCl, 2 mM EDTA) for 2 hr, after which MgCl_2_ was added to a final concentration of 30 mM. GST-WT or GST-S399D was diluted in GEF dilution buffer (20 mM Tris-HCl, pH 7.5, 100 mM NaCl, 20 mM MgCl_2_) along with GTP. To start the exchange reaction, 20 μL of BODIPY-GDP-loaded RhoA and 20 μL GEF+GTP were mixed. The final concentrations after mixing were 0.9 μM for RhoA-BODIPY-GDP, 500 nM for GST-WT or GST-S399D, and 20 μM for GTP. EDTA was added as a positive control for complete nucleotide dissociation and the reference for data normalization. Fluorescence (ex470/em515) was recorded on an Analyst HT plate reader for 45 min at 10 s intervals. The initial reading for each sample was designated as 100%. Five independent experiments were fitted into an exponential decay model using R studio. EDTA plateau readings were defined as 100 % exchange, and the lowest value of each sample was normalized to EDTA and determined % of nucleotide exchange. Groups were compared with the Student t-test.

### Quantification and statistical analysis

Methods of quantification are described within the assays. Statistical analysis was performed with Excel and R. Experimental results were expressed as mean ± SEM. For comparison of normalized data to the reference, one-sample t test was performed. For comparison between two normalized data, paired t-test was performed when the sample size was equal. For samples with unequal sample size a Linead Mixed-Model analysis followed by Tukey test was performed for pairwise comparison. Two-way analysis of variance (ANOVA) followed by Tukey test was performed when comparing data with two independent variables. The specific tests used are indicated in figure legends. Only p values < 0.05 (considered significant) are displayed on the graphs in figures.
