# Supplementary material for: Regulation of Rho guanine nucleotide exchange factor 3 by phosphorylation in the PH domain
Source: iScience. 2025 May 26;28(6):112753. doi: 10.1016/j.isci.2025.112753 (PMC12178798; doi:10.1016/j.isci.2025.112753)
Supplement: Document S1. Figures S1–S7 [file mmc1.pdf]

iScience, Volume *28*

Supplemental information

Regulation of Rho guanine nucleotide exchange factor 3 by phosphorylation in the PH domain

Jesus F. Moreno, Jae-Sung You, Carlos C. Rodriguez, Shashank Pant, Adriana Reyes-Ordoñez, Reean Abdullah, Nilmani Singh, Maxine J. van der Donk, Emad Tajkhorshid, and Jie Chen

SUPPLEMENTAL FIGURES

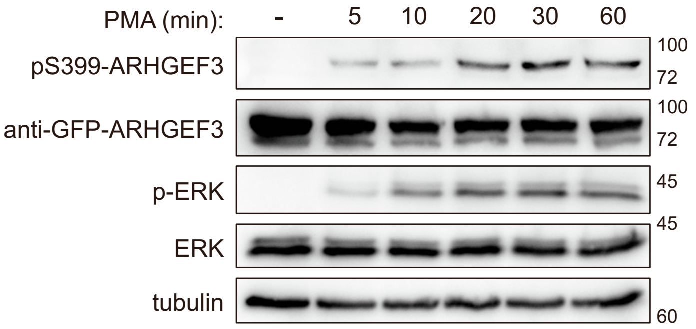

Fig. S1. Time-dependent stimulation of pS399 by PMA. HEK293 cells were transfected with GFP-ARHGEF3 for 24 hr, and stimulated with 100 nM for 5, 10, 20, 30 and 60 min. Cell lysates were analyzed by western blotting. Molecular weight markers (kDa) are indicated on the right side of blots.

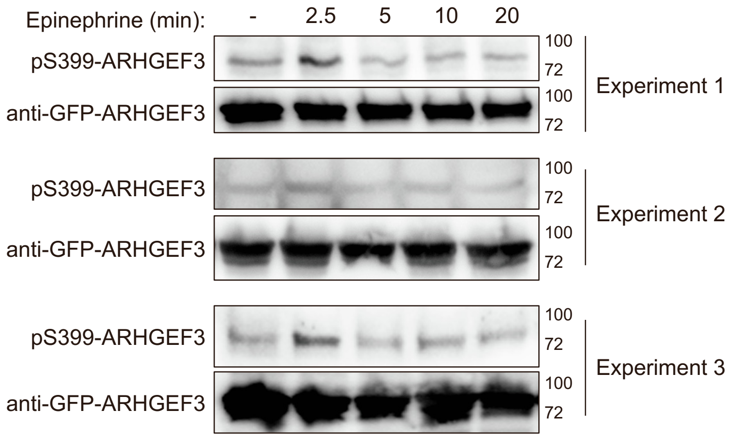

Fig. S2. Epinephrine acutely promotes pS399 phosphorylation. HEK293 cells were transfected with GFP-ARHGEF3 for 24 hr, serum-starved overnight, and stimulated with epinephrine (10 uM) for 2.5, 5, 10, and 20 min. Cell lysates were analyzed by western blotting. Molecular weight markers (kDa) are indicated on the right side of blots.

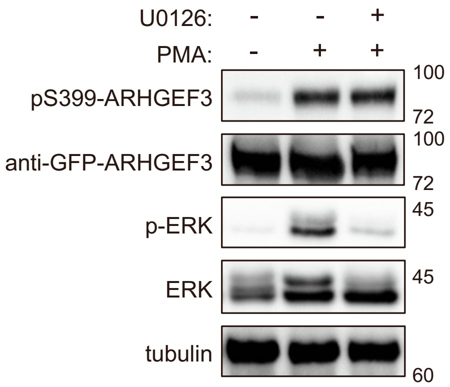

Fig. S3. PMA-stimulated phosphorylation of S399 is independent of ERK. HEK293 cells were transfected with GFP-ARHGEF3 for 24 hr, serum-starved overnight, and stimulated with 100 nM PMA for 30 min. U0126 (5 μM) was added to the cells 10 min prior to PMA stimulation. Cell lysates were analyzed by western blotting. Molecular weight markers (kDa) are indicated on the right side of blots.

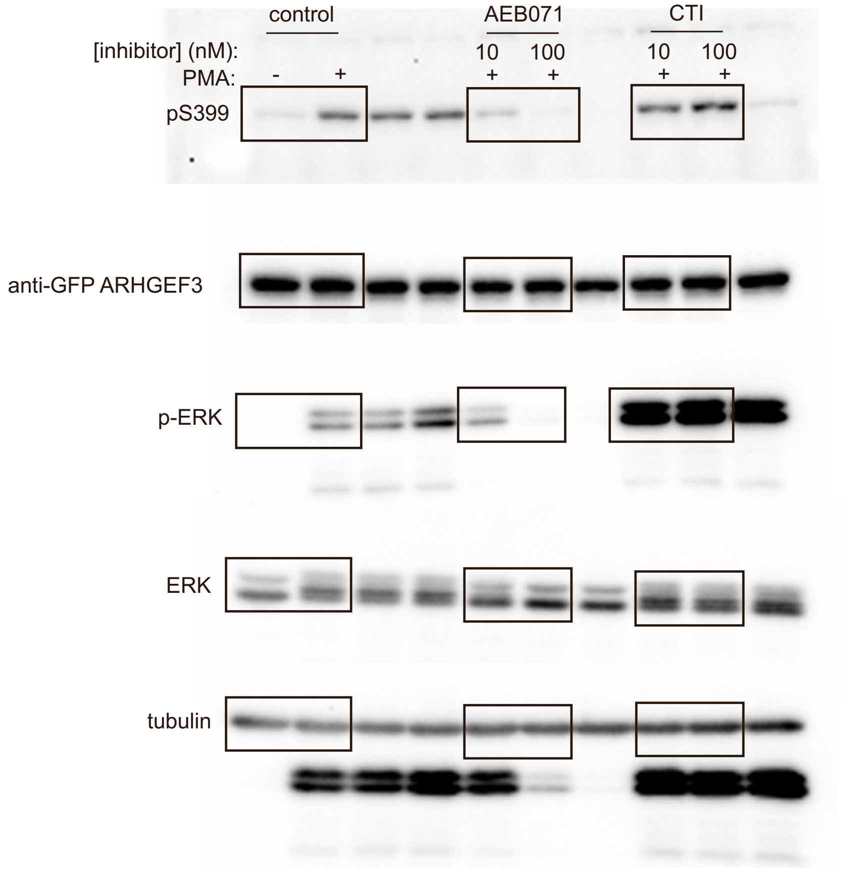

Fig. S4. ARHGEF3 is phosphorylated by nPKC. Full images of western blots for data in Fig. 4D are shown. The bands displayed in the main figure are indicated by boxes.

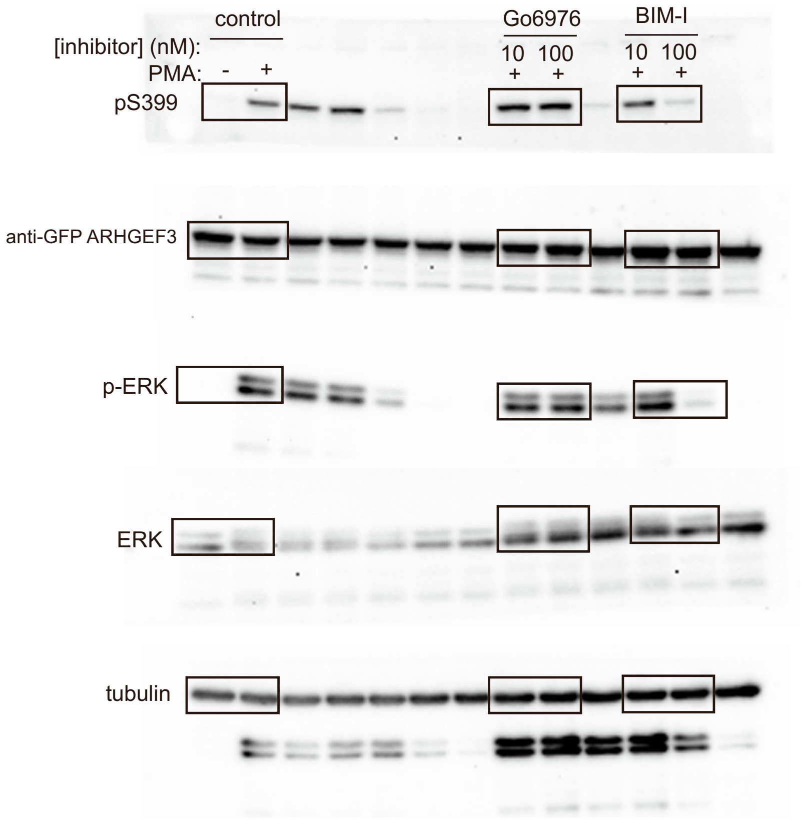

Fig. S5. ARHGEF3 is phosphorylated by nPKC. Full images of western blots for data in Fig. 4D are shown. The bands displayed in the main figure are indicated by boxes.

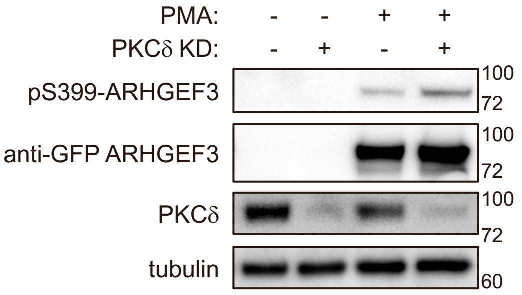

Fig. S6. PKCδ knockdown does not reduce PMA-dependent S399 phosphorylation. HEK293 cells were transduced with shScramble and shPKCδ lentiviruses for 24 h, selected with puromycin (1.5 μg/mL) for 4 days, and then transfected with GFP-ARHGEF3 overnight. Cells were stimulated with 100 nM PMA for 30 min prior to lysis. Cell lysates were analyzed by western blotting. Molecular weight markers (kDa) are indicated on the right side of blots.

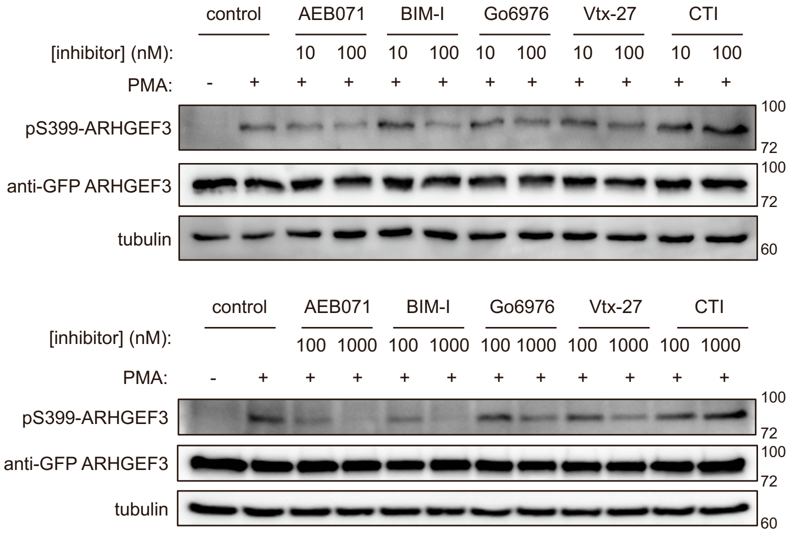

Fig. S7. Effects of PKC inhibitors on PMA-stimulated S399-ARHGEF3 phosphorylation in C2C12 cells. Cells were transfected with GFP-ARHGEF3 for 24 hr, pretreated with AEB071, BIM-I, Go6976, Vtx-27 or CTI at various concentrations for 10 min, and then stimulated with 100 nM PMA for 30 min. Cell lysates were analyzed by western blotting**.** Molecular weight markers (kDa) are indicated on the right side of blots.
